# Osteopathic Manipulative Treatment in Neonatal Intensive Care Units

**DOI:** 10.3390/medsci8020024

**Published:** 2020-06-24

**Authors:** Luca Cicchitti, Alessandra Di Lelio, Gina Barlafante, Vincenzo Cozzolino, Susanna Di Valerio, Paola Fusilli, Giuseppe Lucisano, Cinzia Renzetti, Marco Verzella, Maria Chiara Rossi

**Affiliations:** 1 A.I.O.T-Traditional Osteopathy Italian Academy, 65125 Pescara, Italy; gina.barlafante@aiot.edu (G.B.); vicoz@aiot.edu (V.C.); cinzia.renzetti@aiot.edu (C.R.); or marco.verzella@aiot.edu (M.V.); 2 CORESEARCH-Center for Outcomes Research and Clinical Epidemiology, 65124 Pescara, Italy; dilelio@coresearch.it (A.D.L.); lucisano@coresearch.it (G.L.); rossi@coresearch.it (M.C.R.); 3 Neonatal Intensive Care Unit, Santo Spirito Hospital, 65126 Pescara, Italy; susanna.divalerio@ausl.pe.it (S.D.V.); paolafusilli00@gmail.com (P.F.)

**Keywords:** osteopathy, neonatology, weight change, length of stay, newborn usual care

## Abstract

The aim of this study was to assess the impact of osteopathic manipulative treatment (OMT) on newborn babies admitted at a neonatal intensive care unit (NICU). This was an observational, longitudinal, retrospective study. All consecutive admitted babies were analyzed by treatment (OMT vs. usual care). Treatment group was randomly assigned. Between-group differences in weekly weight change and length of stay (LOS) were evaluated in the overall and preterm populations. Among 1249 babies (48.9% preterm) recorded, 652 received usual care and 597 received OMT. Weight increase was more marked in the OMT group than in the control group (weekly change: +83 g vs. +35 g; *p* < 0.001). Similar trends were found in the subgroup of preterm babies. A shorter LOS was found in the OMT group vs. the usual care group both in overall population (average mean difference: −7.9 days, *p* = 0.15) and in preterm babies (−12.3 days; *p* = 0.04). In severe preterm babies, mean LOS was more than halved as compared to the control group. OMT was associated with a more marked weekly weight increase and, especially in preterm babies, to a relevant LOS reduction: OMT may represent an efficient support to usual care in newborn babies admitted at a NICU.

## 1. Introduction

Worldwide, the estimated number of newborn babies is 136 million, among those, 9.6% (95% CI 9.1; 10.1) is preterm. Prematurity is defined as the birth before 37 weeks of gestational age (GE). Prematurity rate varies across continents, with a higher incidence rate in Africa and Asia, where 85% of all cases is observed [1,2]. Data from industrialized countries show that the prematurity trend of the last decade is slightly declining, particularly in the United States, with a rate of 11.4% in 2013: The same decline is observed in Europe, though rates are markedly lower (5–9%) [3,4]. In Italy, the percentage of preterm births is 7% [5]. Prevalence of premature births may be categorized based on gestational age in weeks: 5% at 28 weeks or less (extreme prematurity), 15% at 28–31 weeks (severe prematurity), 20% at 32–33 weeks (moderate prematurity) and 60–70% at 34–36 weeks (low prematurity) [3].

Premature babies are characterized by higher rates of neurologic, respiratory, cardiovascular, metabolic, psychologic, and cognitive diseases in the first five years after birth [6,7]. It is also shown that these patients are at increased risk of chronic diseases in adulthood [8,9]. Moreover, low-birth-weight contributes to 60% to 80% of all neonatal deaths [1]. According to guidelines, the main criteria for premature baby discharge are the resolution of the clinical conditions related to body temperature, weaning, oxygenation, reaching of oral autonomous feeding, and a positive trend of weight gain [10]. These conditions are indicators of the maturation status of the baby. 

The average length of stay (LOS) in a neonatal intensive care unit (NICU) is 1.9 days for full term babies and ranges from 29.9 days to 64 days for babies with moderate to severe prematurity, respectively [11]. The average cost for admission to a NICU ranges from 1334$ to 32,153$ for term babies and babies with moderate or severe prematurity, respectively [12]. Still, it is evident that the need of healthcare assistance and the hospitalization costs increase as gestational age decreases [10]. Hence, LOS represents one of the main outcomes for the health status of a premature baby, as well as a direct indicator of healthcare costs [13].

Currently, in several Italian hospitals, osteopathic manipulative treatment (OMT) is an integrated activity for the care of babies admitted in the NICU, in spite of robust evidence about its efficacy being lacking. In some randomized clinical trials, OMT was documented to be effective on outcomes such as LOS, weight gain, vomiting, and evacuation as compared to usual care [14,15,16,17], but further research is needed to clarify the impact of OMT on this population. Real-world evidence and use of big data are recognized as a fundamental complement to demonstrate the replicability of experimental data in the real-world [18]. The importance of our study lies in the efficient use of data routinely collected on a large number of babies admitted in the NICU over time and the opportunity to compare data obtained in a real-world vs. experimental setting. Furthermore, primary end-point of the previously conducted RCTs (Randomized Clinical Trials) was the length of hospital stay, but this parameter refers to organizational aspects of the center. Therefore, it could be more appropriate to focus future research on clinical benefits of OMT to understand the level of maturation of the babies promoted by OMT and understand physiologic mechanisms influenced by OMT.

Given these premises, the aim of this study was to assess the impact of OMT on clinical and physiological parameters and LOS as compared to usual care in a large cohort of newborn babies admitted in a NICU by retrospectively analyzing the large clinical database used in the center.

## 2. Materials and Methods

This was an observational, longitudinal, retrospective study. All consecutive babies hospitalized at the NICU of Santo Spirito Hospital in Pescara (Italy) from September 2008 to November 2016 were eligible for the study. A chart review for this retrospective analysis was performed. Starting from all patients registered in the clinical database, the following exclusion criteria were applied: HIV or birth from sieropositive mother; transferred from or to other hospital; genetic or congenital disease; oncologic babies; cardiovascular, urinary, or hematologic abnormalities; suspect or overt necrotizing enterocolitis or abdominal obstruction; birth trauma; surgical patients; pneumoperitoneum; atelectasis.

All babies in the cohort were followed-up from admission to discharge and divided into two groups based on the routine practice. In fact, osteopaths were present in the NICU on two specific days a week and consecutive babies admitted in the NICU in those days were treated with OMT in addition to routine care (OMT group); all other babies admitted in those days when osteopaths were not present in the center served as a control group (usual care group). The usual care administered in the NICU were minimizing parent/infant separation; reducing environmental stressors; protecting sleep; feeding; skin to skin care (SSC); pain and stress management; caregiving interactions; and positioning of infants [19]. The discharge criteria adopted in the NICU were maintenance of body heat at room temperature; coordinated sucking, swallowing, and breathing while feeding; sustained pattern of weight gain; and stability of cardiorespiratory function (no episodes of apnea/bradycardia for 2–5 days, free of supplemental oxygen support) [20].

All data collected during the hospitalization was recorded two times a week on an ad-hoc electronic clinical database routinely used in the NICU. The collected data included:

- baseline information, i.e., personal and socio-demographic information of the baby and the parents, diagnosis at admittance (disease classification based on ICD9-CM standard code), delivery type, and clinical data of the baby at birth (e.g., weight, length, cranial circumference, milk intake);
- follow-up information, i.e., weight, length of stay, spontaneous stool, regurgitation, bilious stasis, blood stasis, gastric vomiting.

The study protocol was approved by the local ethics committee.

### 2.1. Osteopathic Intervention

Osteopathic medicine is a complementary and alternative medicine that uses manual techniques to treat somatic dysfunctions and improve the patient’s health (ICD-10 code: M99.0-99.9). Osteopathic interventions were divided in two steps. The first step was the structural examination to locate the somatic dysfunctions. Tissue texture abnormalities and tone, areas of asymmetry and misalignment of bony landmarks, and the quality of motion, its balance and organization were evaluated. The second step consisted in the osteopathic treatment. The term OMT currently encompasses more than twenty types of osteopath-performed manual treatments [21]. In treating children during the very first days of life, osteopaths can use a wide variety of therapeutic manual techniques to increase range of motion, to improve physiological function and/or support homeostasis that has been altered by somatic dysfunction. The OMT techniques chosen for treating these babies were myofascial release, balanced ligamentous/membranous tension, indirect fluidic, and v-spread [22,23].

The osteopathic structural examination and treatment were performed by registered osteopaths with experience in the neonatology field. Manipulations were administered twice a week during the whole hospitalization period with an average duration of 20 min for each session.

### 2.2. Statistical Methods

Analyses were performed in the overall cohort of hospitalized babies and in the subgroup of preterm babies (gestational age at birth 27–36 weeks). Preterm babies were further stratified by three clinically relevant classes of gestational age at birth (27–31, 32–33, 34–36 weeks). Baseline characteristics were expressed as mean and standard deviation or frequency and percentage for continuous and categorical variables, respectively, and compared using Mann–Whitney or chi-square test, as appropriate. Diagnoses at admission were identified based on the ICD9-CM codes and reported if they had a prevalence >2% in the overall cohort. The distribution of the same diagnoses was also assessed in the subgroups of preterm babies. Frequencies were compared between study arms through Poisson regression analysis.

The primary end-point was the weight gain. The secondary end-points were reduction of length of stay and rate of spontaneous stool, regurgitation, bilious stasis, blood stasis, and gastric vomiting during the hospitalization. Weight change from admission to discharge over time was assessed using mixed models for repeated measurements, with study arm as a factor and time as a covariate, and an autoregressive correlation structure: mean estimated weekly rate of change, as well as between-groups contrast, is provided along with its 95% confidence I\interval (95% CI). Length of stay was estimated through adjusted linear regression models: results are expressed as means and mean changes with their 95% CIs. Rates of spontaneous stool, regurgitation, bilious stasis, blood stasis, and gastric vomiting were estimated with Poisson regression models and expressed as number of events/person-year: results are expressed as incidence rates (IRs) and incidence rate ratios (IRRs) along with their 95% CIs.

The *p*-values < 0.05 were considered statistically significant. All analyses were performed using SAS software release 9.4 (SAS Institute, Cary, NC, USA).

## 3. Results

Overall, 1249 babies recorded in the clinical database were analyzed, of whom 652 received the NICU routine care, and 597 received OMT in addition to routine care. There were 611 preterm babies, representing 48.9% of the cohort, of whom 315 received the NICU routine care and 296 received OMT in addition to routine care. Table 1 shows baseline babies’ characteristics. In the overall cohort, statistically significant differences between groups emerged in terms of cranial circumference, length, and proportion of twins at delivery. The same differences were found in the preterm babies. Adjusted longitudinal models were applied to take into account these differences. Nevertheless, since proportions of twin births were strongly unbalanced among the subgroups and by study arm, twins were excluded from the subsequent analyses to avoid potential confounding. 

**Figure 1:**
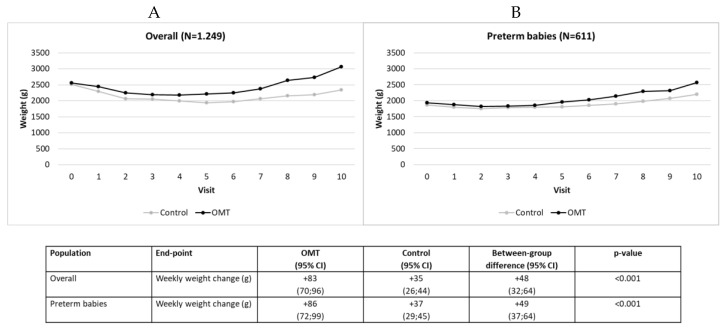
(**A**) Weight change over time in the overall cohort. (**B**) Weight change over time in the subgroup of preterm babies.

In the overall cohort, the two study groups did not show statistically significant differences in body weight at baseline (Table 1). During the follow-up, the OMT group increased in weight more markedly than the control group (mean estimated weekly change: +83 g vs. +35 g; *p* < 0.001 (Figure 1A). Similar trends were found in the subgroup of preterm babies (+ 86 g vs. +37 g; *p* < 0.001) (Figure 1B).

Furthermore, no statistically significant difference emerged in terms of LOS in the overall population, though one was found in the preterm babies (−12.3 days; *p* = 0.04) (Table 2). With the multivariate regression model adjusted for study arm, gestational age, sex, weight, cranial circumference at birth, and milk intake at admission in the NICU, the between-group difference in the length of stay was reduced and an interaction between study arm and gestational age emerged. LOS showed the largest benefits from OMT vs. control in preterm babies born at 27–31 weeks; in this subgroup, LOS was more than halved in comparison with the control group (27.5 vs. 61.3 days; *p* = 0.002), while no effect was documented in the other subgroups (Table 2).

Babies treated with OMT had 14% higher likelihood of spontaneous stool and 43% lower likelihood of blood stasis; on the other hand, they also showed a 91% higher likelihood of bilious stasis (Table 3). In preterm babies, a doubled likelihood of bilious stasis and 86% lower likelihood of blood stasis was found in the OMT vs. the control group (Table 3).

The median (interquartile range) number of OMT sessions was 2 (1–3) in the overall population and 3 (2–5) in the preterm subgroup. Among the preterm babies, the median (interquartile range) number of OMT sessions was 7 (4–9) in the gestational age (GA) 27–31 weeks subgroup, 4 (3–5) in the GA 32–33 weeks subgroup, and 2 (1–3) in the GA 34–36 weeks subgroup.

## 4. Discussion

A cohort of 1249 consecutive premature babies admitted in the NICU of Pescara was analyzed to assess the impact of OMT vs. routine care on many different clinical endpoints. First of all, a positive impact of OMT was found on body weight gain during the admission. After the physiologic weight loss, the rate of weekly weight increase was significantly higher in the OMT group. Remarkably, the gap between OMT and control group widened over time. Moreover, the study shows some benefits of OMT in terms of length of stay. Gestational age plays an important role on the between-group difference in the LOS. It was not considered as a confounder, but rather as a treatment modifier. A statistically significant difference of over one month of admission was found between very premature babies (gestational age 27–31 weeks) treated with OMT vs. control group, while no statistically significant differences emerged in the other subgroups. Additional clinical benefits of OMT emerged in terms of rates of spontaneous stool and blood stasis on the overall sample; on the other hand, a doubled likelihood of bilious stasis was found. These effects of OMT are plausibly mediated by an improved trophotrophic activity, which is in turn due to parasympathetic activity. Actually, preterm babies exhibit reduced heart rate variability [24] and blood pressure variability [25] at term equivalent age, suggesting an altered maturation of the autonomic nervous system. Maturation of the autonomic nervous system is then accompanied by an increase of the parasympathetic activity, described as trophotropic, since it takes part in nourishment [26]. After OMT, results on enhanced bilious vomiting could be due to a parasympathetic functional reactivity that may be responsible for an increased intestinal motility of the first digestive tract, causing duodenal-gastric reflux. Other available gastric findings regarding clotting gastric bleeding that may be due to ooze microbleeding caused by peripartum or delivery stress [27]. In addition, neonatal stress is also due to higher cortisol levels in blood [28]. OMT may be effective in reducing and balancing neonatal stress thereby decreasing gastric bleeding.

A previous multicenter randomized trial showed that OMT can reduce LOS by almost 4 days (−3.9; 95% CI −5.5; −2.3) per premature baby [29] as compared to usual care. A recent systematic review (including a small number of studies) documented an average reduction of −2.7 (95% CI −4.0; −1.4) days in LOS with an average cost reduction per patient of 1545 € (95% CI −1888, −1203) [30]. Our study emphasized that the reduction in LOS is more marked in the real-world than in RCTs, and the entity of the difference is strictly related by the level of prematurity, with OMT particularly beneficial in the GA 24–31 weeks subgroup. Furthermore, this is the first study documenting a positive impact of OMT on weight gain [14,29], while, to the best of our knowledge, no other study assessed the impact of OMT on the other considered endpoints. In addition, it could be underlined that in the previous studies the primary end-point was the LOS, but it is strongly influenced by the organizational characteristics of the centers. Therefore, the weight gain could represent a harder end-point and it should represent the primary end-point in future research.

The study has strengths and limitations. The main strength is the large population involved, which was representative of the whole practice of the center. Moreover, in the absence of randomization procedures, the random allocation of babies in the two groups was naturally ensured by the day of baby admission (i.e., day in which osteopaths were or were not present in the NICU). The major limitation is the retrospective design, in spite of the adjustments for all available possible confounders. Additional limitation is represented by the lack of information about other potential confounders, e.g., interventions used in spontaneous births that may affect the anatomical structure or physiological function or the APGAR score (Appearence–Pulse–Grimace–Activity–Respiratory effort) at birth. On the other hand, the absence of a placebo group is not considered as a bias, since, reasonably, newborns are not exposed to a placebo effect. Available scientific literature does not have enough proof to demonstrate when the placebo effect starts, although it is related to brain development and potentially to expectation or other brain mechanisms [31].

In conclusion, this study documented that compared to usual care, OMT is associated to a more marked weekly weight increase and an improved trophotrophic activity, which is in turn due to parasympathetic activity. The study also confirmed the LOS reduction seen in RCTs and showed also more marked decline in LOS in the real-word vs. the experimental setting. Considering these results, osteopathy has proven to be an efficient support of conventional medicine in newborn routine care.

## Figures and Tables

**Table 1:** Baseline characteristics by treatment (OMT vs. usual care) overall and in preterm babies.

		Overall	Overall Cohort			Preterm Babies		
Variable	Category		Treated (OMT)	Non Treated	*p*-Value	Treated (OMT)	Non Treated	*p*-Value
N. Group		1249	597	652		296	315	
Gestational age (weeks)		36.1 ± 3.5	36.2 ± 3.4	36.0 ± 3.6	0.78	33.3 ± 2.3	32.9 ± 2.6	0.09
Cranial circumference (cm)		299.6 ± 82.7	308.1 ± 71.5	291.5 ± 91.3	**0.03**	282.5 ± 79.5	268.7 ± 91.4	**0.04**
Length (cm)		431.5 ± 120.0	445.4 ± 100.8	418.5 ± 134.3	**0.008**	404.6 ± 110.8	384.1 ± 130.6	**0.04**
Weight (g)		2576.6 ± 859.5	2598.2 ± 852.0	2556.6 ± 866.6	0.53	1996.1 ± 603.5	1923.4 ± 643.0	0.17
Gender	Female	571 (45.7)	271 (45.4)	300 (46.0)	0.83	133 (44.9)	149 (47.3)	0.56
	Male	678 (54.3)	326 (54.6)	352 (54.0)		163 (55.1)	166 (52.7)	
Type of delivery	Spontaneous	460 (36.8)	218 (36.5)	242 (37.1)	0.98	70 (23.6)	76 (24.1)	0.80
	Emergency cesarean section	271 (21.7)	130 (21.8)	141 (21.6)		31 (10.5)	28 (8.9)	
	Elective cesarean section	518 (41.5)	249 (41.7)	269 (41.3)		195 (65.9)	211 (67.0)	
Diagnosis (ICD9-CM)	Twin pregnancy, both born alive	59 (4.7)	36 (6.0)	23 (3.5)	**0.05**	33 (11)	18 (5.7)	**0.02**
	Unspecified prematurity, immaturity, low birth weight	509 (41)	240 (40)	269 (41)	0.77	230 (78)	252 (80)	0.75
	Neonatal respiratory distress syndrome	286 (23)	122 (20)	164 (25)	0.08	88 (30)	111 (35)	0.23
	Transient tachypnea of newborn	62 (5.0)	35 (5.9)	27 (4.1)	0.17	6 (2.0)	4 (1.3)	0.47
	Unspecified perinatal jaundice	223 (18)	104 (17)	119 (18)	0.73	70 (24)	81 (26)	0.61
	Anemia	98 (7.8)	45 (7.5)	53 (8.1)	0.71	41 (14)	48 (15)	0.65
	Neonatal hypoglycemia	53 (4.2)	24 (4.0)	29 (4.4)	0.71	9 (3.0)	11 (3.5)	0.76
	Neonatal hypocalcemia and hypomagnesemia	44 (3.5)	22 (3.7)	22 (3.4)	0.77	8 (2.7)	6 (1.9)	0.52
	Septicemia	40 (3.2)	20 (3.4)	20 (3.1)	0.78	4 (1.4)	7 (2.2)	0.43
	Fever	36 (2.9)	17 (2.8)	19 (2.9)	0.95	5 (1.6)	4 (1.4)	0.81
Milk intake at admission (cc)		143.0 ± 152.5	144.3 ± 151.5	141.8 ± 153.6	0.34	86.5 ± 108.8	82.9 ± 90.2	0.27

Osteopathic manipulative treatment (OMT). Data are mean ± standard deviation or frequency (N and %). Text in bold expresses statistically significant differences.

**Table 2:** Impact of OMT vs. usual care on LOS. Results of linear regression models.

Population	Treatment	N	Mean Estimate (95% CI) *	Mean Estimated Difference (95% CI) *	*p*-Value *	Mean Estimate (95% CI) ^†^	Mean Estimated Difference (95% CI) ^†^	*p*-Value ^†^
Overall	OMT	584	26.0 (18.1; 33.9)	−7.9 (−18.5; 2.7)	0.15	25.8 (18.2; 33.5)	−5.2 (−15.6; 5.1)	0.32
	Control	506	33.9 (26.8; 40.9)	−	-	31.1 (24.2; 37.9)	-	-
Preterm babies	OMT	243	26.4 (17.4; 35.4)	**−12.3 (−24.0; −0.5)**	**0.04**	29.2 (22.3; 36.0)	−2.1 (−11.2; 7.1)	0.65
	Control	282	38.7 (31.0; 46.3)		-	31.3 (25.4; 37.1)		-
GA 27–31 weeks	OMT	53	37.0 (7.3; 66.8)	**−39.4 (−74.0; −4.8)**	**0.005**	27.5 (7.9; 47.0)	**−33.8 (−55.6; −12.0)**	**0.002**
	Control	77	76.4 (58.8; 94.1)	-	-	61.3 (48.2; 74.4)	-	-
GA 32–33 weeks	OMT	60	35.1 (20.1; 50.0)	9.1 (−11.5; 29.7)	0.46	27.5 (7.9; 47.0)	10.3 (−8.5; 29.1)	0.29
	Control	54	26.0 (11.7; 40.2)	-	-	24.7 (11.4; 38.0)	-	-
GA 34–36 weeks	OMT	130	20.8 (10.5; 31.1)	−3.1 (−17.1; 10.9)	0.67	20.1 (11.8; 28.4)	4.7 (−6.8; 16.2)	0.42
	Control	151	23.9 (14.4; 33.4)	-	-	24.8 (15.9; 33.8)	-	-

Osteopathic manipulative treatment (OMT); length of stay (LOS); gestational age (GA); confidence intervals (95% CI); interquartile (IQ); * model adjusted by treatment; † model adjusted by treatment, sex, weight, milk intake, and cranial circumference. Text in bold expresses statistically significant differences.

**Table 3:** Other clinical end-points. Results of Poisson models.

	Group	Overall Number of Episodes	% of Patients with at Least 1 Episode	Distribution of Episode (Median and Range)	IR per Person-Year (95% CI)	IRR (95% CI)
(A) Overall sample (N = 1249)						
Spontaneous stool	OMT	4411	98	7.0 (0.0–54.0)	262.0 (243.1; 282.3)	**1.14 (1.03; 1.27)**
	Control	5129	97	6.0 (0.0–71.0)	229.3 (213.8; 245.8)	
Regurgitation	OMT	95	12	0.0 (0.0–5.0)	5.82 (4.7; 7.2)	0.96 (0.72; 1.29)
	Control	145	15	0.0 (0.0–8.0)	6.0 (5.0; 7.3)	
Bilious stasis	OMT	135	4.2	0.0 (0.0–86.0)	8.9 (7.1; 11.2)	**1.91 (1.34; 2.73)**
	Control	92	5.3	0.0 (0.0–11.0)	4.6 (3.5; 6.1)	
Blood stasis	OMT	18	2.2	0.0 (0.0–5.0)	1.2 (0.9; 1.7)	**0.57 (0.39; 0.85)**
	Control	41	3.8	0.0 (0.0–6.0)	2.1 (1.7; 2.6)	
Gastric vomiting	OMT	167	21	0.0 (0.0–9.0)	9.2 (7.5; 11.3)	0.88 (0.68; 1.15)
	Control	226	23	0.0 (0.0–10.0)	10.5 (8.9; 12.4)	
(B) Preterm babies (N = 611)						
Spontaneous stool	OMT	2565	99	8.0 (0–49.0)	223 (202–246)	1.08 (0.95–1.23)
	Control	3422	98	9.0 (0–71.0)	205 (189–223)	
Regurgitation	OMT	57	14	0 (0–5.0)	5 (4–7)	1.02 (0.71–1.47)
	Control	87	18	0 (0–8.0)	5 (4–6)	
Bilious stasis	OMT	121	6.2	0 (0–86.0)	11 (8–15)	**2.31 (1.47–3.64)**
	Control	77	8.5	0 (0.11.0)	5 (3–7)	
Blood stasis	OMT	3	1.2	0 (0–1)	0.3 (0.1–0.6)	**0.14 (0.07–0.30)**
	Control	31	5	0 (0–6)	1.9 (1.5–2.4)	
Gastric vomiting	OMT	102	26	0 (0–9)	9 (7–11)	1.14 (0.82–1.58)
	Control	128	29	0 (0–10)	8 (6–10)	

Osteopathic manipulative treatment (OMT); Incidence Rate (IR); Incidence Rate Ratio (IRR); confidence intervals (95% CI). Text in bold expresses statistically significant IRR.
